# A proof-of-concept study poised to remodel the drug development process: Liver-Chip solutions for lead optimization and predictive toxicology

**DOI:** 10.3389/fmedt.2022.1053588

**Published:** 2022-12-16

**Authors:** Zaher Nahle

**Affiliations:** Center for Contemporary Sciences, Gaithersburg, Maryland, MD, United States

**Keywords:** predictive toxicology, organ-chip, liver-chip, drug development, microphysiological systems (MPS), FDA regulations, animal testing, drug-Induced liver toxicity (DILI)

## Introduction

In a key study published on December 6, 2022, in *Nature Communications Medicine,* Ewart et al*.* introduced a paradigm-shifting solution to the inefficient drug discovery process (1). It entails the incorporation of Liver-Chip platforms as refinement tools within the preclinical assessment and lead optimization stages. This work first appeared in bioRxiv and is now entitled “*Performance assessment and economic analysis of a human Liver-Chip for predictive toxicology*.”

Today, there are fundamental challenges in the process of pharmaceutical drug development. According to the National Center for Advancing Translational Sciences (NCATS), one of 27 Institutes and Centers at the National Institutes of Health (NIH), “Therapeutic development is a costly, complex and time-consuming process. The average length of time from target discovery to approval of a new drug is about 14 years. The failure rate during this process exceeds 95 percent, and the cost per successful drug can be $2 billion or more.” Such failure rate can be attributed, in part, to the dearth of “human relevance” among the experimental models used in drug screening and the modeling of human pathologies—challenges that have burdened the pharmaceutical industry for decades and contributed to its “productivity crisis” (2, 3). In fact, an objective examination of the many artificial animal models used to study humans reveals disparate characteristics—with hardly any capturing the sequela of human diseases. Radical structural, physiological, anatomic, digestive, genomic, metabolic and behavioral differences underlie those discrepancies among species.

Ewart et al*.* is ostensibly the largest systematic investigation evaluating the advantage of using human Liver-Chips to predict drug toxicity in humans, a critical step in drug development. Briefly, the study showed, using a panel of characterized drugs, that the Liver-Chip correctly identified 87% of agents causing drug-induced liver injury in humans. Furthermore, the Liver-Chip did not falsely label any safe drug tested as toxic—in essence preventing the premature jettison of useful drugs. Such outcomes are superior to those produced with standard preclinical packages for hepatic toxicity, including routine cell culture techniques, 3D hepatic spheroids, or animal models.

The findings were articulated through large comparative analyses coupled with methodical economic assessments. The latter focused on evaluating the productivity gains acquired by adopting the Liver-Chip platforms in preclinical stages of drug development. In doing so, Ewart et al*.* tackled at once stubborn challenges in pharmaceutical medicine like safety, efficacy, productivity, and cost. To that end, the solutions presented in the study could alter industry practice for the foreseeable future. As such, they merit the attention of the scientific community and FDA regulators. Here, we highlight the key findings of the study. We also discuss the implication of the work in the context of the growing demand for human relevancy across the preclinical stages of drug development, and the expanding field of Micro-Physiological Systems.

## Key findings

**Figure 1:**
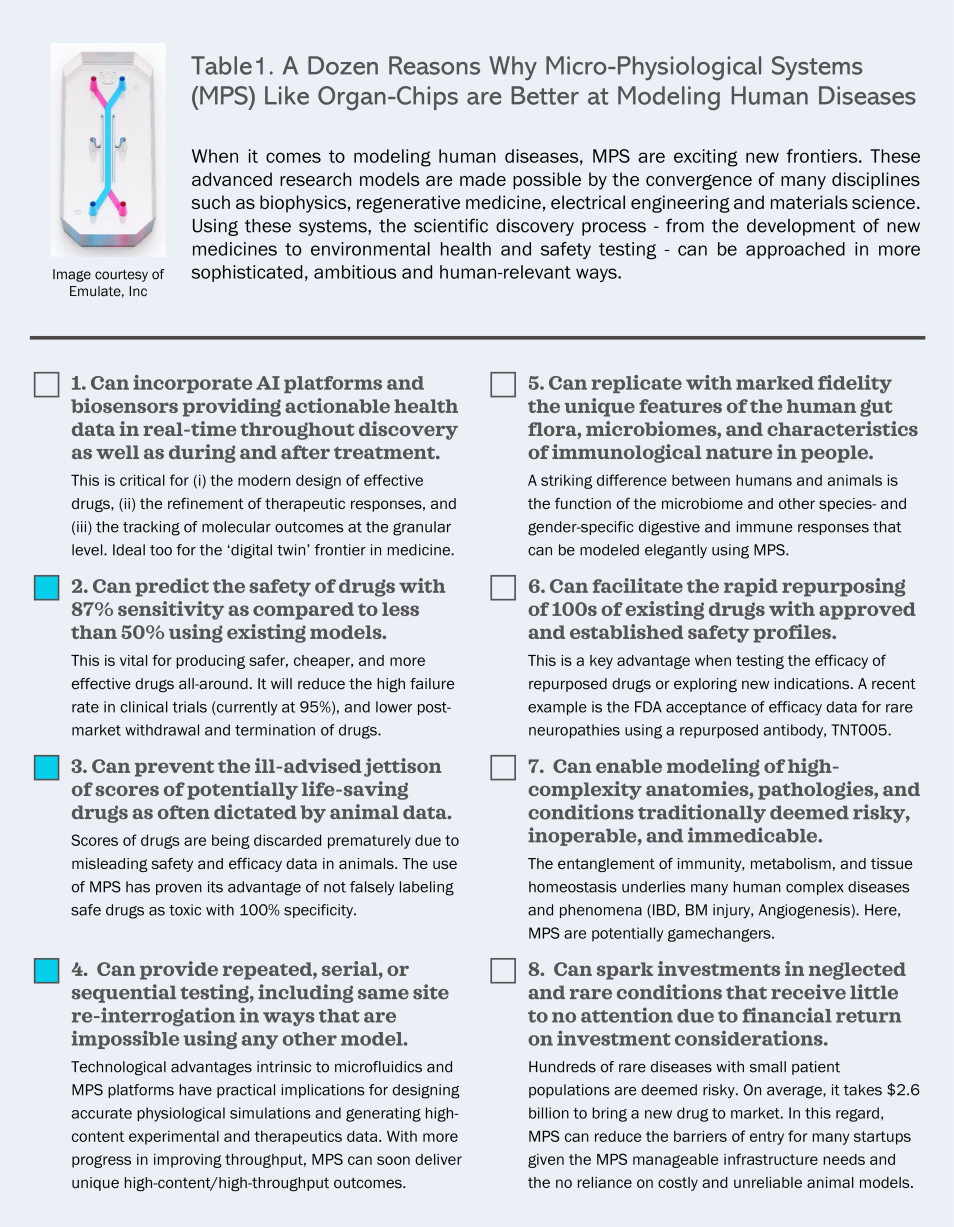

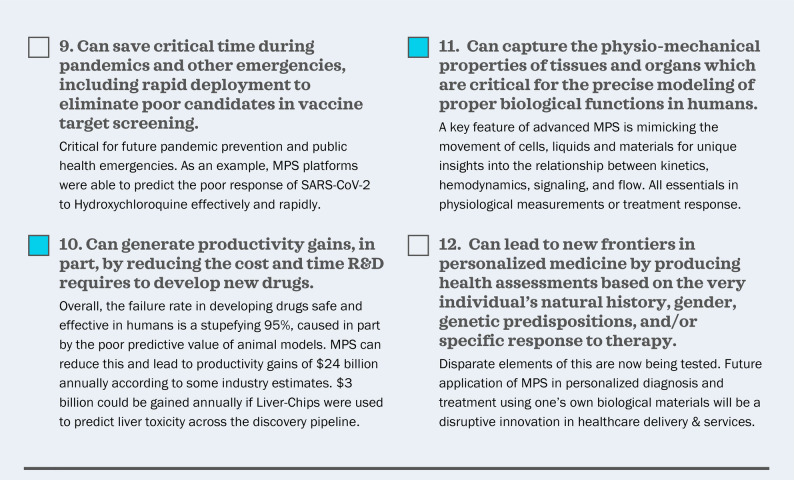
A dozen reasons why micro-physiological systems (MPS) like organ-chips are better at modeling human diseases. AI: Artificial Intelligence; IBD: Inflammatory Bowel Disease; BM Injury: Bone Marrow Injury. Filled squares represent aspects directly related to Ewart et al., reviewed in this opinion article.

Micro-Physiological Systems, or MPS, are experimental tools and platforms engineered to capture with fidelity, cell-to-cell communication, fluid dynamics, physio-mechanical features as well as other structural, dimensional and functional parameters of a physiological system for research and discovery purposes. Their value as consolidated investigative models is made possible by progress in fields like biophysics, regenerative medicine, materials science and electrical engineering. Among the most popular MPS are Organ-Chip platforms [7]. These platforms experienced rapid growth over the last decade, both in the number of their developers and users, as well as the applications they offer. On the market today are Chips designed to study specific organs such as lung, liver, skin, heart, kidney, intestine as well as a multitude of disease conditions, biological phenomena, immunity and public health matters (4–58). Organ-Chip platforms continue to grow in their sophistication and appeal as useful research tools. They are being steadily incorporated into all aspects of scientific investigations—from biomedical research to environmental health and safety testing—in academia, industry and governmental agencies. A checklist summarizing the broad appeal of MPS, with reference to the main findings of Ewart et al*.,* is provided in Figure 1.

Ewart et al*.* takes advantage of the Emulate Liver-Chip system and focuses on liver toxicity. In the first part, the study sought to establish that the Liver-Chip system satisfies the strict quality guidelines of the Innovation and Quality (IQ) MPS consortium. IQ MPS is an affiliate of the International Consortium for Innovation and Quality in Drug Development, a collaboration of pharmaceutical and biotechnology companies that seeks to advance the use of MPS in drug discovery (59). The guidelines set stringent standards to determine whether a Liver-Chip model replicates the functions of the liver, including Drug Induced Liver Injury (DILI)—also known as drug-induced hepatoxicity, injury to the liver that is caused by medication. In the second part, research was conducted to demonstrate the improved sensitivity of the human Liver-Chip for DILI prediction compared to two other established systems used in preclinical predictive toxicology, namely animal models and cell culture tools known as spheroids. In the third part, data was presented to show that the specificity of the Liver-Chip-mediated drug test could be further improved by accounting for relevant biological interactions, namely drug-protein binding. Finally, the authors concluded by addressing the economic value of incorporating the Liver-Chip as predictive toxicity models in preclinical decision making.

Below, are some aspects of the study that we find particularly notable. These include the focus on liver toxicity, superior capability for predicting drug toxicity, productivity gains and the quality control performed.

### First, the focus on liver toxicity

The study focuses on hepatic predictive toxicology. In the United States and Europe, toxicity failure is the main cause of withdrawn and discontinued drugs. Liver toxicity in particular is the most common toxicity type associated with drug withdrawal (21%), followed by cardiovascular (16%), hematological (11%), neurological (9%), carcinogenicity (8%), and others (35%) (60–62). Therefore, understanding drug-induced liver toxicity is a worldwide priority. Beyond drug discovery, the work on liver toxicity has broad applications for diagnostics and testing since DILI can be caused by a range of agents including Anesthetics, Non-Steroidal-Anti-Inflammatory Drugs (NSAIDS), Antimicrobial medications, Antibiotics, Antifungal agents, Anti-viral drugs, oral Hypoglycemics, Lipid-lowering drugs, and certain Herbal and Traditional medicines.

### Second, the marked sensitivity demonstrated in predicting the toxicity of drugs

Arguably, the most striking feature of the study is the discovery that the Liver-Chip can predict with at least 80% sensitivity the accurate toxicity status of known toxic drugs. Specifically, the Liver-Chip was able to correctly flag as toxic 12 out of 15 drugs included in the battery of drugs tested to evaluate the Liver-Chip performance. Of note, the study examined 18 drugs (15 hepatotoxic) across three hepatocyte donors. The roster of toxic drugs is comprised of those found to induce liver toxicity clinically after progressing to first-in-human administration. Notably, each toxic drug tested was historically evaluated using animal models and, in every case, the toxic drug was deemed to have an acceptable therapeutic window qualifying it to advance into clinical trials. In this context, the ability of the Liver-Chip to flag 80% of the toxic drugs for their DILI risk and predict their toxicity with such accuracy is a remarkable step forward. Importantly, data provided in the study show that the sensitivity of the Liver-Chip can be further increased from 80% to 87% through technical adjustments accounting for drug-protein binding.

### Third, the superior capability to not falsely label safe chemicals as toxic

One of the most consequential decisions in the drug development process is the labeling of an experimental drug as either safe or toxic following preclinical assessments. This is typically done using preclinical toxicology studies involving animal models. A toxic classification almost certainly leads to the abandonment of the agent being tested. In this regard, the Liver-Chip does not mislabel any safe drug as toxic, hence performed with a 100% specificity in testing drugs for DILI risk. This superior performance is a sharp contrast to existing methodologies where this type of mislabeling is common. Published data for 3D hepatic spheroids shows a sensitivity rate of 42% and a specificity rate of 67%.

### Fourth, the convincing economic argument presented

A central theme of the study by Ewart et al*.* is the economic argument articulated within. Liver-Chip and other Organ-Chips could lead to billions annually in productivity gains for Research and Development (R&D) if incorporated routinely in the preclinical assessment and lead optimization stages of drug development. Using an economic value model of drug development that incorporates decision quality (63, 64), the study demonstrates that $3 billion could be gained annually if Liver-Chips were used to predict liver toxicity across the discovery pipeline. If other types of organ toxicities (four key toxicity sites modeled in the study) could be predicted by Organ-Chips, assuming similar performance compared to the Liver-Chip, the technology could generate over $24 billion annually in R&D productivity gains.

### Fifth, the rigorous quality control performed

Noted is the work performed to establish the physiological relevance of the human Liver-Chip model related to hepatic conditions, both structurally and functionally. Structural, morphological, and functional analysis were performed using a combination of advanced methodologies. Several types of microscopy systems (light, confocal, and electron) were used to test the Liver-Chips. This combination provides in-depth qualitative and quantitative data to establish the presence of three-dimensional structures and the cell-to-cell interaction seen in actual liver tissues. In addition, known relevant markers of functional liver cells (Albumin and Urea production) were tested successfully for cellular functionality of the Liver-Chip. For drug toxicity assessments, clinically relevant markers were also used [Albumin production inhibition and the increases in release of alanine aminotransferase (ALT) protein, used clinically as a measure of liver damage], alongside other morphological assessments and established markers of cell death, such as Caspase 3/7 (65). A blinded set of 27 drugs with known hepatoxic and nontoxic behavior were analyzed in the study. The performance of 870 human Liver-Chips across the blinded set that incorporated cells from three human donors for a period of 16 weeks were tested to determine that the Liver-Chip replicated human biological responses.

### Sixth, the limitation of the study

Some challenges, both practical and conceptual, are still limiting the use of Organ-Chips in general. These include the inability to incorporate the host immune core components, the limited integration of several organs simultaneously, the absence of Common Data Elements (CDEs), universal standards and performance criteria, especially among different Chips by different manufacturers, the awareness of the proper context of use of these platforms, the access to relevant and well characterized cell sources, access to training or MPS core facilities, the bias for existing models due to familiarity, nonspecific binding caused by some materials used in the fabrication of Organ-Chips and the uncertainty of regulatory acceptance for MPS-generated data. The latter is further examined in the discussion section.

In the current study, a limitation of the initial data set was the use of cells from two human donors only. Notably, when data from a third donor were incorporated in the subsequent revision of the paper, no reduction in the marked sensitivity of the drug test was observed, further bolstering the veracity of these Liver-Chip platforms as credible and reproducible tools. That said, a limitation to the Liver-Chips used is their inability to assess at this time the toxicity of agents that function through complex immune-mediated toxicity, a particular form of toxicity that requires additional elements and response time beyond the testing capacity of these Chips. This could change in the future with the development of MultiSystem/MultiOrgan-Chips that bring added sophistication to these platforms. Another inherent limitation is the material used in constructing this Organ-Chip [Polydimethylsiloxane (PDMS)], that could lead to non-specific binding. Advances in materials science would mitigate such limitation in the future.

It is important to note that the reason why 90% of clinical drug development fails is a complex, multifactorial process. Indeed, the examination of clinical trial data from 2010 to 2017 reveals four main reasons attributed to such high failure rate: lack of clinical efficacy (40%–50%), unmanageable toxicity (30%), poor drug-like properties (10%–15%), and lack of commercial needs and poor strategic planning (10%) (66–68).

Here, Ewart et al*.* introduced a powerful tool to address a major challenge and one of the leading causes of unmanageable toxicity, hepatic toxicity. Succinctly, the Liver-Chip outperforms existing models that are based on widely used preclinical toxicology packages. In addition, the specific feature of this Liver-Chip—not falsely labeling safe chemicals as toxic—could very well confer the most benefit going forward. To the best of our knowledge, such fidelity is unprecedented. The Organ-Chip approach will help prevent the abandonment of potentially useful and safe drugs early in the process. In this context, it is hard to assess the extent of lost opportunities produced from erroneously discarding drug candidates too early. But examining old data obtained prior to the requirement from regulatory agencies to use animal models for preclinical assessment is very telling in this regard. Many beneficial and life-saving drugs like penicillin (fatal to guinea pigs), aspirin (embryo toxicity in rats and rhesus monkeys), and paracetamol (toxic in cats and dogs) would have failed standard preclinical toxicity testing in animals and therefore could never have reached the market. Finally, even if a fraction of the monetary value derived from the productivity gains of incorporating Organ-Chips in predictive toxicology is realized, this would still translate into safer, cheaper, faster, and more effective medications. All in all, the findings of the study support, at a minimum, the incorporation of Liver-Chip platforms in the initial stages of drug screening—a step that shall inform better decision-making in pharmaceutical medicine.

## Discussion

This study is one of the most critical developments in the field of Organ-Chip technology. It shows the primacy and utility of the Liver-Chip platforms in predictive toxicology. It also demonstrates the immediate readiness of such technology to transform critical phases of the drug development process, in particular lead optimization and preclinical assessment, making the entire process safer, cheaper, faster, and more effective. It is important to avoid extrapolation of these results to unsuitable contexts. At this juncture, one must highlight the practical application of the Liver-Chip within defined points of integration in the lead optimization stages, a notion underscored by the authors in a representative schematic (Ewart et al*.* Figure 5).

In addition to its scientific merit, Ewart et al*.* must be examined in the context of the value it adds to advancing the public good. A stupefying 90%–95% of experimental drugs fail in humans mainly due to efficacy (40%–50%) or safety (30%) concerns. That is despite these drugs having good efficacy and safety profiles in animal models granting them regulatory clearance to proceed into clinical trials. In addition, scores of potentially life-saving drugs are prematurely discarded due to the ill-advised jettison based on animal data or traditional *in vitro* toxicity packages. This leads to significant productivity loss, delays in producing vital pharmaceuticals and exorbitant cost that is ultimately passed onto consumers. This work represents the most compelling interdisciplinary (scientific and economic) argument that Organ-Chip platforms for predictive toxicology can far outperform traditional methods, including *in vivo* animal models.

Notably, Ewart et al*.* is taking place in a climate of legislative momentum seeking to advance research methodologies that are based on human biology. As such, the study is a driver for policy change that prioritize human-relevant research methods in the regulatory acceptance process. Notably, in the United States (U.S.), the FDA Modernization Act of 2021 (H.R.2565) passed in the U.S. House of Representatives in June 2022, and in September 2022 the very similar FDA Modernization Act 2.0 (S.5002) passed unanimously in the U.S. Senate (69, 70). These legislations seek to amend the outmoded regulatory guidance at the FDA—they broaden the options for drug developers seeking regulatory approvals to include new technologies and human-relevant testing methods like Organ-Chips, *in lieu* of animal experimentation. In addition to Organ-Chips and MPS, alternative methods are broadly defined. They include sophisticated computer modeling and artificial intelligence (AI) as well as advanced 3D bioprinting techniques like those using bioinks of living cells to replicate natural features of human tissues.

The FDA has taken some steps to explore the value of alternative methods like MPS, including Organ-Chips, in preclinical regulatory approvals (71). A new initiative was conceived by the FDA “to implement a cross-agency New Alternative Methods Program.” In its budget proposal for fiscal year 2023, the agency also expressly stated that, “New alternative methods have the potential to provide both more timely and more predictive information to accelerate product development and enhance emergency preparedness.” In addition, the agency co-hosted an inaugural MPS World Summit earlier in 2022. The FDA also sustains programs like the Predictive Toxicology Roadmap and ISTAND with somewhat similar objectives. Recently, the FDA accepted efficacy data generated through MPS to support the authorization of a clinical trial, setting a precedent in authorizing Investigational New Drug (IND) applications using, in part, alternative methods. The said trial is investigating a repurposed antibody (TNT005) with previously established safety profiles.

Of note, health agencies worldwide are experiencing a growing demand for human-relevant approaches and testing methods. For instance, U.S. lawmakers proposed in 2019 plans to reduce primate research at the National Institutes of Health (72). That same year, the U.S. Environmental Protection Agency (EPA) chief pledged to eliminate all mammal testing by 2035. In a signed memo, EPA chief asserted that “The EPA will reduce its requests for, and our funding of, mammal studies by 30 percent by 2025 and eliminate all mammal study requests and funding by 2035” (73). Moreover, The Humane Research and Testing Act of 2021 (HRTA, H.R.1744) was introduced in the U.S House of Representatives in 2021. This legislation seeks to establish the National Center for Alternatives to Animal Research and Testing within the National Institutes of Health with the sole purpose of “(1) developing, promoting, and funding alternatives to animal research and testing; and (2) developing a plan for reducing the number of animals used in federally funded research and testing.” That is in addition to the introduction of The Humane and Existing Alternatives in Research and Testing Sciences Act (HEARTs, H.R. 4101) and The Animal Freedom from Testing, Experiments, and Research Act of 2021 (AFTER, S.1378). Importantly, The Humane Cosmetics Act of 2021 was also introduced in the U.S. Senate (S.3357) and U.S. House of Representatives (H.R.6207). It is an ambitious legislation that “generally prohibits animal testing in the evaluation of cosmetic products, and it prohibits the sale or transport of cosmetics developed using animal testing, subject to civil penalties.”

A similar momentum is taking place in Europe. As an example, a landmark resolution was approved by the European parliament in September 2021. Such measure provides the policy framework for the European Commission to phase out the use of animals for all scientific purposes. In short, the legislation sought to implement changes across a wide range of sectors including funding, enforcement, implementation, education, training, industry relations, regulatory affairs, and private sector engagement. It is referred to as B9-0425/2021, “A Motion for a European Parliament resolution on plans and actions to accelerate the transition to innovation without the use of animals in research, regulatory testing and education.”

It is against this backdrop that the progress made in the Ewart et al*.* study is poised to disrupt the existing drug development paradigm. Such progress ushers a new era where disease modeling that is based on human relevance is an attainable, realistic priority. Ewart et al*.* and similar future work from the scientific community provide the impetus for investigators, lawmakers, regulators, investors and industry leaders to seriously consider the potentially significant benefits of making adjustments to the existing regulatory process. That is in addition to changes to the discovery pipeline infrastructure, taking advantage of Organ-Chip platforms as top-hole, human-relevant tools in pharmaceutical medicine.

## Author contributions

ZN conceived, designed, wrote, and corresponded the work. ZN (*znahle1@jh.edu*) is matriculated at the Johns Hopkins Carey Business School MBA program. His efforts in developing the manuscript were enabled by access to school resources, including academic libraries.

## Conflict of interest

The authors declare that the research was conducted in the absence of any commercial or financial relationships that could be construed as a potential conflict of interest.
